# Asthma is associated with atherosclerotic artery changes

**DOI:** 10.1371/journal.pone.0186820

**Published:** 2017-10-26

**Authors:** Izabela Tuleta, Dirk Skowasch, Florian Aurich, Nicolas Eckstein, Robert Schueler, Carmen Pizarro, Nadjib Schahab, Georg Nickenig, Christian Schaefer, Simon Pingel

**Affiliations:** Department of Internal Medicine II–Cardiology, Pulmonology and Angiology, University of Bonn, Bonn, Germany; Beijing Key Laboratory of Diabetes Prevention and Research, CHINA

## Abstract

Asthma is a chronic airway inflammation with a potential systemic impact. Atherosclerosis is a chronic inflammatory artery disease. The aim of our study was to prove if there is a correlation between the occurrence of asthma and increased atherosclerotic vessel disorders. Vessel status was compared between mild-to-moderate, severe allergic asthma and matched controls. Measurements of artery stiffness were calculated by central pulse wave velocity, ultrasonographic strain imaging and ankle-brachial index. Atherosclerotic plaque burden was assessed by colour-coded duplex sonography. Additionally, analysis of cardiovascular and asthma blood markers was conducted. Arterial stiffness expressed as an increased central pulse wave velocity and decreased circumferential and radial strains as well as the prevalence of media sclerosis were significantly higher among asthma patients compared to controls. Atherosclerotic plaque burden was relevantly increased in asthma groups vs. controls (severe asthma: 43.1%, mild-to-moderate asthma: 25.0%, control: 14.3% of study participants). Except for the elevated IgE and fibrinogen concentrations as well as leukocyte number there were no relevant differences in the blood parameters between the groups. Allergic asthma is associated with distinct atherosclerotic artery changes compared to the respectively matched control collective. The severity of asthma correlates with more pronounced pathological vessel alternations.

## Introduction

Asthma is a chronic inflammatory disease of the airways. A growing body of evidence shows that asthma may affect also extrapulmonal tissue. Patients with asthma demonstrate an increased risk for cardiovascular complications [1]. Moreover, asthma is associated with functional (higher central pulse wave velocity, [2]) and structural (increased intima-media-thickness, [3]) vessel changes. However, data linking asthma with atherosclerosis are not consistent [4]. The pathomechanisms underlying asthma-related atherosclerotic arterial alternations are not clear. Some studies reported on phenomena occurring in asthma such as inflammation [5], infection [6], oxidative stress [3] or activation of blood platelets [7] as potential mechanisms leading to the acceleration of atherosclerosis in this patients`collective. Not only asthma itself, but also the antiasthmatic drugs may have an influence on the vessel status. Specifically, application of oral cortisone in patients with severe asthma may have deleterious effects on the vasculature [8]. In contrast, inhaled cortisone used as a controller medication in mild-to-moderate asthma may act protectively on the vascular system [9]. Against this background, the aim of our present work was to evaluate functional and structural atherosclerotic vessel changes in patients with mild-to-moderate and severe asthma compared to control individuals. Additionally, we aimed to determine the levels of cardiovascular risk markers in blood.

## Material and methods

### Patients`characteristics

The research was conducted according to the principles of the Declaration of Helsinki and was approved by the local ethics committee at the Medical Faculty of the University of Bonn. Written informed consent has been obtained from all subjects. The study has been conducted between October 2015 and July 2016. A total of 82 consecutive outpatients with allergic asthma have been enrolled in the study during control visits in our pneumological department. Allergic asthma was defined as an increase in forced expiratory volume in 1 second (FEV1) of more than 12% following administration of short acting ß2-agonist or a decrease in FEV1 of more than 200ml in bronchial challenge test with methacholine and detection of at least three allegro-allergens in specific IgE blood measurements or in skin prick test. All patients presented stable disease without clinical exacerbation at the time point of the study performance. Patients with immunodeficiency or a history of autoimmune or other than asthma chronic inflammatory diseases were excluded. Asthma patients have been divided into two groups: mild-to-moderate (n = 24) and severe asthma (n = 58). Mild-to-moderate asthma patients were under inhaled therapy with no present or past treatment with omalizumab or oral cortisone. In contrast, severe asthma was characterized by a maximal inhaled therapy combined with a present or past administration of omalizumab and/or cortisone oral. 21 non-asthmatic patients served as controls. There were no significant differences in sex, age, body mass index and cardiovascular risk factors such as arterial hypertension, hypercholesterolemia, diabetes mellitus, nicotine abuse or history of artery disease between these three groups. Lung function showed significantly decreased absolute and predicted FEV1 values as well as significantly increased absolute and predicted total airway resistance values in asthma patients compared to controls. The exact baseline characteristic of the patients and controls is presented in the Table 1.

**Table 1:** Baseline characteristic of asthma patients and controls. Except for a significant reduced lung function and specific antiasthmatic therapy in asthma patients there were no relevant differences between asthma and control groups. BMI = body mass index. FEV1 = forced expiratory volume in 1 second, TLC = total lung capacity, RV = residual volume, Rtot = total airway resistance Data are presented as a mean ± standard error of the mean (SEM) or n (%). P<0.05 = significant.

	Group 1 (Mild asthma, n = 24)	Group 1 (Severe asthma, n = 58)	Group 3 (No asthma = Control, n = 21)	P value
**Sex, male (n, (%))**	7 (29.2)	21 (36.2)	11 (52.4)	0.257
**Age (years)**	46.4±2.2	52.8±1.9	48.3±2.7	0.107
**BMI (kg/m**^**2**^**)**	27.0±0.9	27.4±0.7	25.0±0.6	0.155
**Arterial hypertension (n, (%))**	8 (33.3)	20 (34.5)	9 (42.9)	0.755
**Hypercholesterolemia (n, (%))**	5 (20.8)	6 (10.3)	3 (14.3)	0.411
**Diabetes mellitus (n, (%))**	3 (12.5)	4 (6.9)	0 (0.0)	0.264
**Nicotine abuse**				0.110
- Current (n, (%))	5 (20.8)	2 (3.4)	3 (14.3)	
**-** Former (n, (%))	3 (12.5)	12 (20.7)	2 (9.5)	
**History of coronary heart disease (n, (%))**	0 (0)	1 (1.7)	0 (0.0)	0.700
**History of peripheral arterial disease (n, (%))**	5 (20.8)	8 (13.8)	1 (4.8)	0.341
**Therapy**				
- Inhaled glucocorticoids n (%)	21 (87.5)	58 (100)	0 (0.0)	<0.001
- Long acting beta-2 agonists = LABA (n, (%))	16 (66.7)	58 (100)	0 (0.0)	<0.001
- Long-acting muscarinic antagonists = LAMA (n, (%))	6 (25.0)	37 (63.8)	0 (0.0)	<0.001
- Oral glucocorticoids (n, (%))	0 (0.0)	20 (34.5)	0 (0.0)	<0.001
- Omalizumab, current (n, (%))	0 (0.0)	47 (81.0)	0 (0.0)	<0.001
- Omalizumab, former (n, (%))	0 (0.0)	8 (13.8)	0 (0.0)	<0.001
**Lung function**				
- TLC (l)	5.7±0.3	6.0±0.2	6.9±0.4	0.018
- TLC (% predicted)	99.9±3.3	101.9±1.8	111.4±4.6	0.074
- FEV1 (l)	2.8±0.2	2.3±0.1	3.3±0.3	0.001
- FEV1 (% predicted)	90.2±4.4	76.6±2.8	100.6±4.0	<0.001
- RV (l)	2.1±0.1	2.7±0.1	2.7±0.2	0.010
- RV (% predicted)	114.0±7.4	135.7±5.4	131.6±9.9	0.080
- Rtot (l*kPa/s)	0.3±0.0	0.4±0.0	0.2±0.0	0.037
- Rtot (% predicted)	105.1±13.4	132.0±13.5	64.7±8.0	0.038

### Angiological measurements

Classical parameters of vessel stiffness have been calculated by means of central pulse wave velocity (cPWV) and ankle-brachial index (ABI). Assessment of central pulse wave velocity (cPWV) was performed using AngE Pro8® (Sonotechnik Austria, Maria Rain, Austria). Ankle-brachial index (ABI), defined as the ratio of the systolic blood pressure (SBP) measured at the ankle to that measured at the brachial artery [10], was calculated for both tibial posterior artery and the dorsal pedis artery on both sides. Mean ABI values >1.3 pointed to an increased vascular stiffness and were considered as an indicator for media sclerosis [11]. Colour-coded duplex sonography was used for the detection of atherosclerotic plaques. Presence of cerebral or peripheral arterial disease was defined by ≥1 atherosclerotic plaque in the duplex sonography.

Ultrasonographic strain imaging included global average circumferential strains (% of vessel circumference changes), global average radial strains (% of vessel wall thickness changes), radial displacement (changes in vessel wall position in mm), global average circumferential strain rates (vessel circumference changes per second) and global average radial strain rates (vessel wall thickness changes per second). Common carotid artery was shown in the two-dimensional short axis (Philips iE33). Four electrocardiography (ECG)-triggered cardiac cycles were stored for the offline analysis. Following creation of a region of interest, the frame-to-frame displacements of the speckles representing tissue movements were analyzed by using of a computer workstation equipped with a speckle-tracking software package (Image Arena Version 4.6, TomTec Systems GmbH, Munich, Germany). This method has been previously described [12, 13].

### Assessment of different blood parameters

The following cardiovascular risk and asthma markers in blood have been examined using standard laboratory tests: total cholesterol, HDL- and LDL-cholesterol, lipoprotein(a), C-reactive protein (CRP), interleukin-6, soluble interleukin-2 receptor (sIL-2R), immunoglobulin E (IgE), fibrinogen, d-dimer, leukocytes, lymphocytes, eosinophils, hemoglobin, thrombocytes.

### Statistical analysis

Mean values of the determined parameters ± standard error of the mean (SEM) were compared by means of ANOVA for three groups and by means of the Student's t-test for two selected groups. Potential differences between categorical variables were calculated by using of the chi-square test. P<0.05 was considered as statistically significant.

## Results

**Fig 1:**
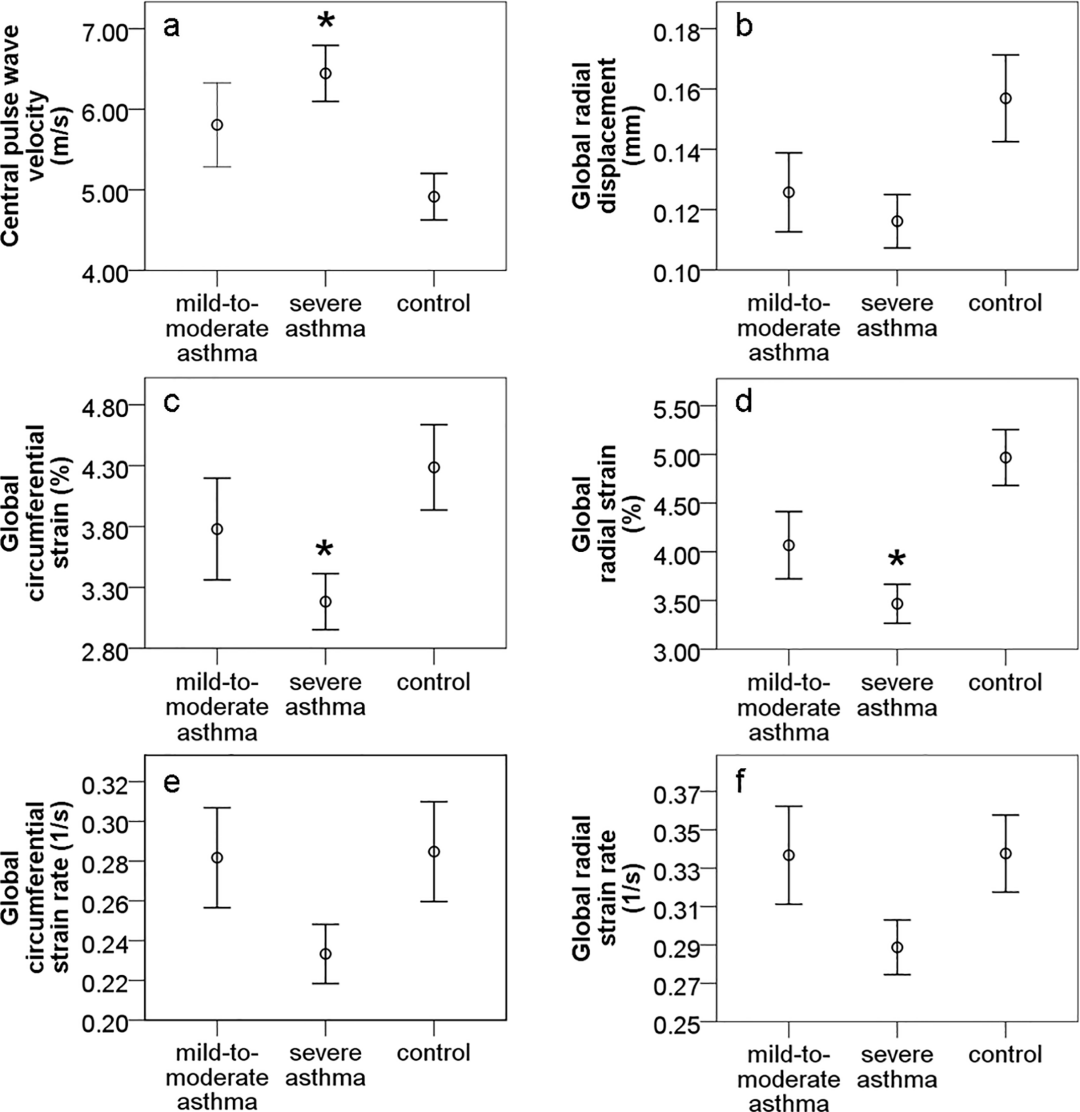
Diagrams representing changes of arterial stiffness measured by central pulse wave velocity (a) and ultrasonographic strain imaging (b-f) depending on the presence of asthma. P<0.05 = significant vs. control.

As a key finding, the angiological results have shown an increased arterial stiffness and more manifest atherosclerosis in patients with asthma, especially in those with its severe form, compared to the baseline matched controls. In detail, central pulse wave velocity differed significantly across the groups studied (mild-to-moderate asthma: 5.81±0.52 m/s, severe asthma: 6.45±0.35 m/s, control: 4.91±0.29 m/s; p = 0.019) with the highest values in severe asthma collective (p = 0.005 vs. control; Fig 1A).

**Fig 2:**
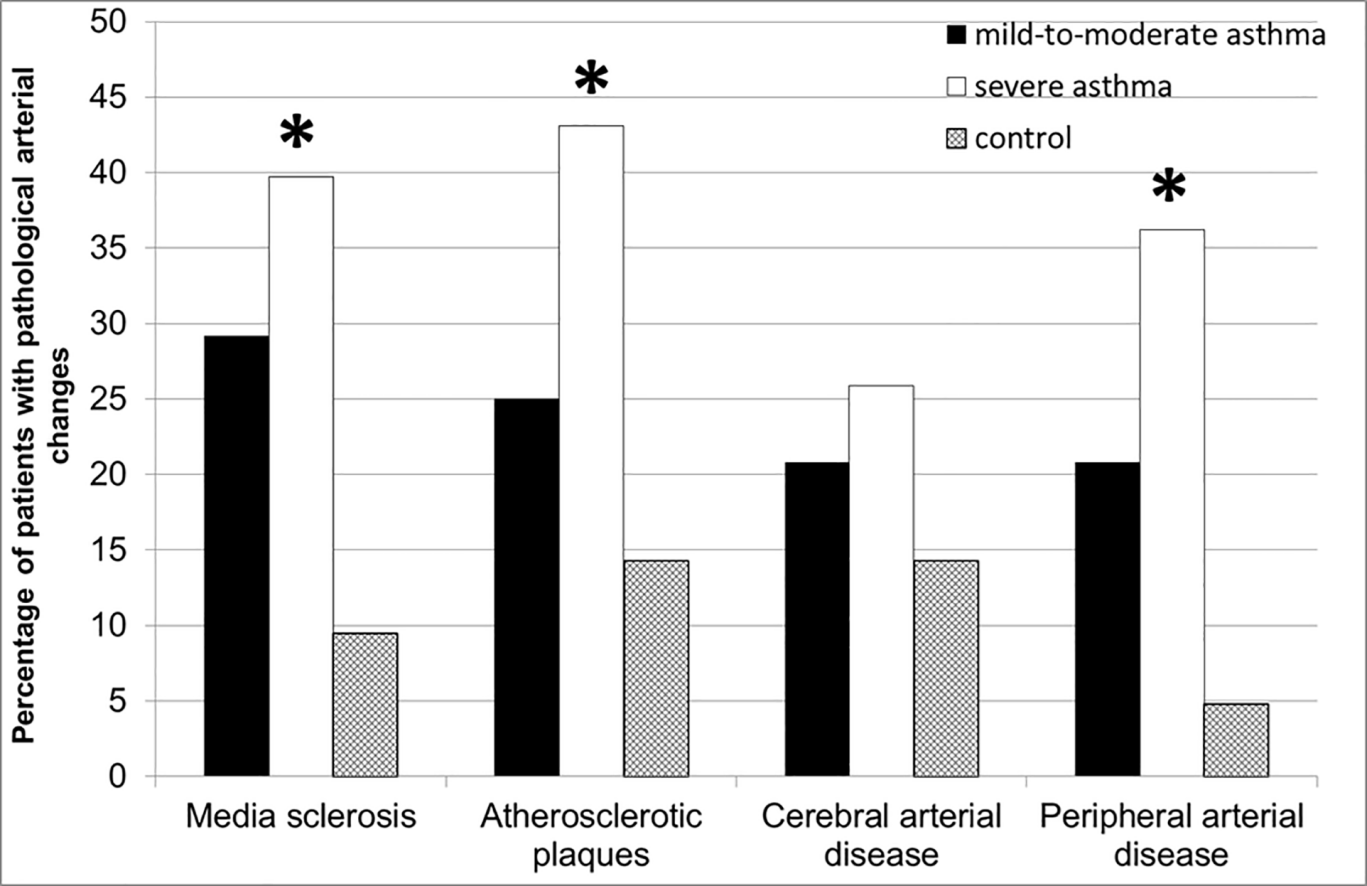
Increased prevalence of media sclerosis, atherosclerotic plaques as well as cerebral and peripheral artery disease in asthma patients compared to respectively matched controls. P<0.05 = significant across the groups.

Vascular strains presented a relevant decrease of circumferential strains and radial strains in severe asthma (3.18±0.23%, 3.47±0.20%, respectively) in comparison to control (4.29±0.35%; p = 0.013, 4.97±0.29%, p<0.001; resp., Fig 1C and 1D). Circumferential and radial strains were also reduced in mild-to-moderate asthma (3.78±0.42%, 4.07±0.35%), but without reaching a statistical significance vs. control (p = 0.363, p = 0.054, resp., Fig 1C and 1D). Other vascular strains, such as: radial displacement, circumferential strain rates and radial strain rates demonstrated no significant differences across the groups (p = 0.055, p = 0.101, p = 0.088, resp.), however there was a trend towards lower values of these parameters in severe asthma (0.12±0.01 mm, 0.23±0.01 1/s, 0.29±0.01 1/s, resp.) compared to control (0.16±0.01 mm, 0.28±0.03 1/s, 0.34±0.02 1/s; Fig 1B, 1E and 1F). Radial displacement tended also to be reduced in mild-to-moderate asthma (0.13±0.01 mm) vs. control (Fig 1B). Circumferential strain rates and radial strain rates showed the same mean values in mild-to-moderate asthma (0.28±0.03 1/s, 0.34±0.03 1/s, resp.; Fig 1E and 1F) as for control. Media sclerosis was more pronounced in severe (n = 39.7%) and mild-to-moderate (n = 29.2%) asthma compared to the non-asthma group (n = 9.5%; p<0.037; Fig 2).

Manifest atherosclerotic vessel changes were significantly more frequent in asthma patients, particularly in severe asthma individuals vs. controls. Specifically, atherosclerotic plaques were seen in 43.1% of severe asthma, 25.0% of mild-to-moderate asthma and 14.3% of control study participants (p = 0.035; Fig 2). Atherosclerotic artery alternations accounted for the predominance of peripheral artery disease in severe asthma (36.2%), followed by mild-to-moderate asthma (20.8%) vs. control (4.8%; p = 0.015; Fig 2). Similarly, cranial artery disease could be diagnosed more often in asthma patients (severe asthma: 25.9%; mild-to-moderate asthma: 20.8% vs. control: 14.3%), however these differences were not statistically significant (p = 0.540; Fig 2).

**Fig 3:**
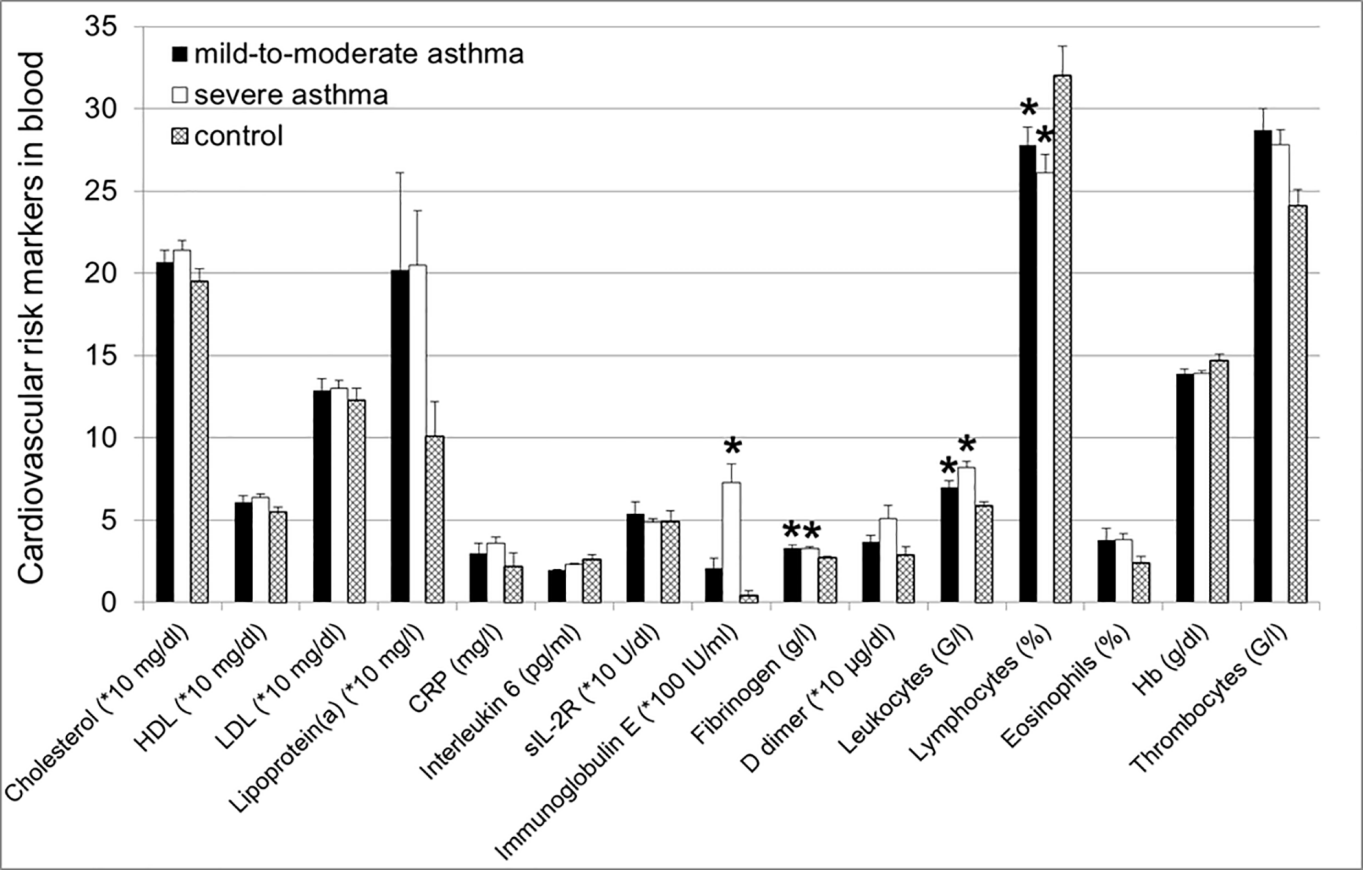
Elevated IgE and fibrinogen levels and higher number of leukocytes with reduced lymphocyte fraction in asthma groups vs. control. No significant differences in total cholesterol, HDL- and LDL-cholesterol, lipoprotein(a), C-reactive protein (CRP), interleukin-6, soluble interleukin-2 receptor (sIL-2R), d-dimer, eosinophils, hemoglobin and thrombocytes between the groups. P<0.05 = significant vs. control.

Next step was to determine if the above described pathological arterial alternations were accompanied by the changes in blood levels of different cardiovascular risk and asthma markers. Our results demonstrated that patients with asthma characterized by higher IgE blood concentrations (severe asthma: 7.3±1.1*100 IU/ml, mild-to-moderate asthma: 2.1±0.6*100 IU/ml, control: 0.4±0.3*100 IU/ml; p = 0.002) showed significant elevated levels of fibrinogen (severe asthma: 3.3±0.1 g/l, mild-to-moderate asthma: 3.3±0.2 g/l vs. control; p = 0.005) and leukocytes (severe asthma: 8.2±0.4 G/l, mild-to-moderate asthma: 7.0±0.4 G/l vs. control; p = 0.001) with a reduced lymphocyte fraction (severe asthma: 26.1±1.1%, mild-to-moderate asthma: 27.8±1.1% vs. control; p = 0.025) and a slightly increased number of eosinophils (severe asthma: 3.8±0.4%, mild-to-moderate asthma: 3.8±0.7% vs. control; p = 0.191) compared to control (2.7±0.1 g/l, 5.9±0.2 G/l, 32.0±1.8%, 2.4±0.4%, resp.; Fig 3).

Additionally, there was a tendency towards higher levels of lipoprotein(a) and thrombocytes in asthma patients (severe asthma: 20.5±3.3*10 mg/dl, mild-to-moderate asthma: 20.2±5.9*10 mg/dl; severe asthma: 27.8±0.9 G/l; p = 0.05, mild-to-moderate asthma: 28.7±1.3 G/l, resp.) compared to controls (10.1±2.1*10 mg/dl; p = 0.280, 24.1±1.0 G/l; p = 0.076, resp.; Fig 3). Interestingly, there were no relevant differences in classical cardiovascular risk factors such as cholesterol, HDL cholesterol, LDL cholesterol or inflammatory markers such as C-reactive protein (CRP) between the groups (Fig 3).

For evaluation of a potential influence of the asthma-associated medicamentous therapy, a subanalysis of patients with omalizumab and oral cortisone treatment (group 1a, n = 16) vs. patients on omalizumab without oral cortisone therapy (group 1b, n = 31) has been conducted. Baseline characteristics were similar in both groups. The only difference was that patients from group 1a had significantly lower FEV1 values in comparison with the group 1b (FEV1: 1.9±0.2 l vs. 2.7±0.1 l, resp., p = 0.002; FEV1 (% predicted): 67.0±5.2% vs. 85.8±3.4%, resp., p = 0.003). Angiological examinations have shown worse vessel status with a significantly higher percentage of atherosclerotic plaques in the group 1a vs. group 1b (central pulse wave velocity: 6.87±0.60 m/s vs. 5.87±0.46 m/s, resp., p = 0.194; global radial displacement: 0.09±0.01 mm vs. 0.12±0.01 mm, resp., p = 0.154; global circumferential strain: 2.62±0.38% vs. 3.46±0.34%, resp., p = 0.141; global radial strain: 3.21±0.37% vs. 3.70±0.29%, resp., p = 0.324; global circumferential strain rate: 0.18±0.02 vs. 0.26±0.02, resp., p = 0.029; global radial strain rate: 0.25±0.02 vs. 0.31±0.02, resp., p = 0.124; atherosclerotic plaques: 68.8% vs. 32.3%, resp., p = 0.017; cranial artery disease: 37.5% vs. 19.4%, resp., p = 0.176; peripheral artery disease: 56.3% vs. 29.0%, resp., p = 0.069). Blood analysis demonstrated except for increased IgE levels and most likely cortisone-associated changes in the white blood cell count in group 1a (IgE: 10.2±1.7*100 IU/ml vs. group 1b: 3.5±0.8*100 IU/ml, p = 0.009; leukocytes: 10.1±0.9 G/l vs. group 1b: 7.4±0.2 G/l, p = 0.001; lymphocytes: 21.7±2.6% vs. group 1b: 28.9±1.2%, p = 0.005) no relevant differences between both groups.

## Discussion

Our data show pathological artery changes in asthma patients compared to the control collective. Specifically, asthma was not only associated with preatherosclerotic vessel alternations, such as higher arterial stiffness, but much more with increased prevalence of manifest atherosclerosis compared to non-asthma individuals. Indeed, asthma has already been described to be a risk factor for stroke and heart diseases [14,15,1]. Generally, an impaired lung function has been associated with an increased cardio- and cerebro-vascular disease risk [16,17]. Increased brachial-ankle pulse-wave velocity has been detected in stable and severe asthma patients [2]. Adult-onset asthma but not child-onset asthma was associated with an augmented carotid intima-media-thickness (IMT) among women but not men [18]. However, it has also been reported on contradictory results with no differences in aortic stiffness parameters in childhood-onset asthma compared to control patients likely due to the anti-inflammatory effect of inhaled steroids [4] or even diminished atherosclerosis in asthmatic patients as a possible consequence of elevated circulating endogenous heparin-like material [19].

As traditional cardiovascular risk factors, such as: arterial hypertension, diabetes mellitus, hypercholesterolemia, obesity and nicotine abuse were similar between asthma and control individuals, we aimed to determine other potential risk markers in the blood. As a result, only IgE and fibrinogen levels as well as leukocyte number and subtypes differed significantly between the groups. Fibrinogen is independently linked to enhanced rates of cardiovascular complications [20]. It has been shown that persistent asthmatics have higher CRP [2] and fibrinogen levels in blood [1]. Leukocytes are the main cells of inflammation which is a hallmark of atherosclerosis [21]. In particular, high neutrophil-lymphocyte ratio (NLR) has a predictive value for future vascular events [22]. In our work we have observed a trend towards elevated lipoprotein(a) concentrations as well as thrombocyte and eosinophil counts. Increased lipoprotein(a) levels are associated with the risk of future cardiovascular disease events [23]. Activated blood platelets, in turn, may promote atherosclerosis [24]. Moreover, peripheral blood eosinophilia is a predictor factor for death from coronary heart diseases and cerebrovascular diseases [25]. Additionally, it has been shown that allergic asthma may accelerate atherosclerosis via activation of Th2 and Th17 cells in apolipoprotein E deficient mice [26]. Inversely, subcutaneous allergen-specific immunotherapy was associated with lower risk for autoimmune disease and acute myocardial infarction, as well as decreased all-cause mortality [27]. Furthermore, asthma is related to the increased arterial inflammation measured by arterial 18F-fluorodeoxyglucose (FDG) uptake on positron emission tomography (PET) images [28]. In line with this result, multiple inflammatory markers known as risk markers for atherosclerosis development were detected in asthma [29]. A positive relationship was also found between increased oxidative stress and carotid intima-media thickness in asthmatic patients [3]. Single nucleotide polymorphisms (SNPs) in some genes in asthma patients may influence levels of serum markers of inflammation and endothelial dysfunction connected to cardiovascular disease progression [30]. Other studies demonstrated further data supporting a thesis that some pathological phenomena such as leukotriene pathways [31], circulating platelet-leukocyte aggregates [32], activation of toll-like receptors (TLRs, [33]), release of platelet activating factor (PAF) from mast cells [34] or infection with *Chlamydia pneumoniae* [6] are common for asthma and atherosclerosis.

In our study, all vessel changes were more pronounced in severe asthma compared to mild-to-moderate asthma. Interestingly, patients on the add-on combined therapy of oral corticosteroid and omalizumab (group 1a) showed even greater atherosclerotic artery alternations than the patients with the same therapy except for oral corticosteroids (group 1b). Since the lung function was more impaired in group 1a vs. group 1b, it is difficult to judge if the severity of asthma, differences in the treatment or both influenced this finding. In this context, the role of antiasthmatic medication in the process of vasculopathy is not clear. Some works reported on the beneficial effects of inhaled glucocorticosteroids on the vessel changes [35]. One could think that oral cortisone due to its antiinflammatory properties, such as a direct inhibition of the expression of adhesion molecules in vascular endothelial cells [36] or of the chemotactic response of monocytes exposed to oxidized beta-VLDL [37] may also have a positive influence on the artery alternations. Contrary to this presumption, the use of oral corticosteroids was linked to an increased risk for myocardial infarction [8]. However, different animal experiments showed inconsistent results ranging from steroid-associated reduction of plaque progression [38], no effect of cortisone [39] to progression of atherosclerosis under cortisone therapy [40]. Inhibition of intracellular glucocorticoid may ameliorate metabolic syndrome and thus treat effectively atherosclerosis. Explanation of this finding could be a multifaceted nature of cortisone which contrary to the generally accepted view that cortisone acts purely anti-inflammatory may also enhance diverse inflammatory processes [41].

In our present study, we did not observe any relevant cortisone-related side effects, such as: increase in blood pressure, induction of hypercholesterolemia or diabetes mellitus. One may assume that asthma-specific activity and adverse effects of a long-term high dose cortisone therapy may have led to the worst vessel status in patients with severe asthma under oral cortisone therapy.

In conclusions, our data implicate an association between asthma, in particular severe asthma, and pathological artery changes, including a significantly increased atherosclerotic plaque load.
